# Balancing selection and candidate loci for survival and growth during larval development in the Mediterranean mussel, *Mytilus galloprovincialis*

**DOI:** 10.1093/g3journal/jkad103

**Published:** 2023-05-13

**Authors:** Zhihan Hua, Nathan Churches, Sergey V Nuzhdin

**Affiliations:** Department of Molecular Biology, University of Southern California, Los Angeles, CA, 90089, USA; Department of Biology, Lecture Faculty, San Francisco State University, San Francisco, CA, 94132, USA; Department of Molecular Biology, University of Southern California, Los Angeles, CA, 90089, USA

**Keywords:** mussels, larval development, genetics, pool-seq, balancing selection, survival, growth

## Abstract

Many marine bivalves have complex life histories with distinct developmental processes and genetic mechanisms. Larval development for most bivalves is often a prolonged and crucial physiological stage, where they suffer mass mortality due to early-acting genetic load. In this study, we describe genetic changes taking place within a single generation of families of the Mediterranean mussel *Mytilus galloprovincialis* over 23 days of larval development. Using replicated cultures and a pooled sequencing approach, we demonstrate that temporal balancing selection at the majority of loci preserve genetic variation in the early developmental stages of *M. galloprovincialis*. Balancing selection may be the mechanism which maintains standing genetic variation within the mussel genome and may improve the chances of survival and shield larvae from high levels of genetic load. Additionally, we used changes in allele frequencies to identify potential size-associated SNPs and viability-associated SNPs and found that patterns of genetic changes in directionally selected SNPs cannot be simply explained by traditional theories of genetic purging or directional selection without consideration of balancing selection. Finally, we observed a negative correlation between larval growth rates and survival, implying a potential trade-off relationship between the 2 commercially relevant phenotypes.

## Introduction

World fisheries are important natural resources, yet many have been overexploited. The end of the 20th century saw a significant decline in global wild fisheries catch (Pauly et al. 2022), and while 21st century wild fisheries that are closely managed remain stable or are experiencing increasing abundance, those that are not scientifically managed are typically over-exploited (Hilborn et al. 2020). Even so, stable or increasing population sizes and scientific management do not necessarily belie stock levels which can support established or targeted maximum sustainable yields: in 9 out of 10 European eco-regions, the majority of fishery stocks are unsustainably exploited (Froese et al. 2018). This landscape of overexploitation is juxtaposed with an ∼7–10% annual increase in consumer demand for seafood (Ruggles 2018). Substantial efforts have been made to find a solution to balance consumer demand and seafood supply and to identify the best tools to manage global fish stock in the future (FAO 2020; Hilborn et al. 2020). Aquaculture has become 1 of the fastest-growing food production sectors in many regions of the world (FAO 2020; Naylor et al. 2021), and the number of domesticated and farmed marine species has also increased in recent decades (Teletchea 2021). Nevertheless, only a limited number of species have reached a high level of domestication in aquaculture (Teletchea 2019), while the majority of aquaculture products remain little different from their wild counterparts (FAO 2020). Genomic selection (GS) is a promising tool in which genome-wide molecular markers are used to develop marker-based models and breeding programs built on genotype-to-phenotype correlations (Goddard and Hayes 2007; Bhat et al. 2016). GS has been successfully implemented in several terrestrial livestock and grains, including poultry (516 g increase in body weight and 140 g in breast muscle weight) (Lee 2021; Tan et al. 2022), cattle (an ∼1% increase in milk production, 40–80 kg/cow/year) (Bouquet and Juga 2013; Wiggans et al. 2016), and rice (40% increase in training population) (Cui et al. 2020). Implementation of GS to improve aquaculture cultivars has also been established for some finfish species, including rainbow trout, *Oncorhynchus mykiss* (Vallejo et al. 2017; Yoshida et al. 2018), Atlantic salmon, *Salmo salar* (Correa et al. 2015; Bangera et al. 2017), and coho salmon, *Oncorhynchus kisutch* (Barría et al. 2018), but it remains relatively understudied in low-trophic level aquaculture organisms (Gutierrez et al. 2018; Gutierrez et al. 2020). Selective breeding programs for food crops and livestock are, by comparison, significantly more advanced, and they can serve as templates for aquaculture production. The domestication of marine species can be a powerful tool to promote a more sustainable development of global aquaculture and to help solve the current problems facing wild fishery exploitation.

Sea-based bivalve aquaculture is a major worldwide industry and is an excellent source of nutrient-rich food (Liu and Ralston 2021), known to be high in energy and proteins and rich in essential minerals (selenium, iodine), vitamins (A, B12, and D), and omega-3 fatty acid (Orban et al. 2002; Schug et al. 2009; FAO 2016; Wijsman et al. 2019). Varieties of oyster, clam, mussel, and scallop are the top 4 most produced bivalves, representing 33%, 37%, 13%, and 17% of annual global landings, respectively (EFSA 2014). They accounted for 16 million tons of marine animal aquaculture in 2015 and represented 15.9% of the total worldwide aquaculture sector, with an estimated market value of $17.1 billion (Elbashir et al. 2018). In addition to their traditional market value and nutritional benefits, bivalves can also act as ecosystem engineers by shaping, controlling, and improving their surrounding environment (Filgueria et al. 2015; Smaal et al. 2019).

Bivalves are highly fecund, yet their offspring can suffer substantial early (day 0–21) mortality: 80–98% type III survivorship events are not uncommon in hatchery culture (Plough et al. 2016; Yin and Hedgecock 2021). Bivalve life history is characterized by a relatively long life [typically several years or decades, though some live for hundreds of years (Butler et al. 2013)], delayed reproduction, and extremely high fecundity—100 s of millions of eggs can be produced annually in mature females of some bivalves (Rickman et al. 2000; Llodra 2002; Winemiller 2005). Although external factors underlying type III survivorship in bivalves have been well studied (e.g. environment and nutrition), genetic factors also play a role and are still not entirely understood (Hjort 1914; Plough 2016).

Bivalve larvae develop into a ciliated cluster of cells within hours after fertilization and reach a “ciliated trochophore” stage ∼24 h postfertilization (hpf), and in some species, this stage is also characterized by the development of a flagellum. This initial stage is followed by the secretion of the first shell material and a distinctive “D” shape (hence commonly referred to as the “D-hinge larvae” stage) and the development of a ciliated feeding and swimming organ, the velum, after which this veliger stage is named. The transition from the trochophore to veliger stage typically occurs around 24–48 hpf and is maintained from 1 to several weeks, depending on the species. For oysters, mussels, and clams, the maximum size during the veliger stage is typically 200–400 *µ*m. The veliger larvae will eventually develop an “eye-spot” and the ciliated foot common to many mollusks (and is now called a pedi-veliger) which can be used for locomotion and allows for the beginning of benthos-associated behaviors. This foot allows the bivalve larvae to seek a suitable place to perform metamorphosis—the transition from free-swimming larvae to a sessile juvenile by secretion of a bioadhesive material or via byssal threads—and it is during this metamorphosis where many commercial hatcheries observe significant mortality episodes. Metamorphosis is 1 of the most important life history events for bivalves, and several transcriptomic studies across many species demonstrate significant expression profile changes at this stage (Niu et al. 2016; Foulon et al. 2019; Hao et al. 2019; Wang et al. 2023). For the Mediterranean mussel, *Mytilus galloprovincialis*, transcriptomic studies focusing on metamorphosis show enrichment of differentiation and biosynthesis transcripts, perhaps at the expense of their health: immune-related transcripts decrease during this stage (Moreira et al. 2018). This reduction in expression of immune-related genes may be a factor in high mortality events, or high mortality could be attributed to the expression of previously silent genes, newly activated at metamorphosis, or from deleterious or nonideal allelic combinations from maternal and paternal backgrounds (i.e. genetic load and/or specific combining ability effects).

One of the long-standing challenges in aquaculture cultivar management is the maintenance of sufficient genetic variability (i.e. reduction of inbreeding depression) in broodstock, while simultaneously achieving genetic improvement of selected populations for a multitude of outplanting environments. To accomplish these commercial breeding goals, genetic contributions to early mortality must be better understood. Many studies have investigated the background genetic load in marine animals (see Plough 2016 for a review), including studies which have utilized inbred lines of Pacific oyster, *Crassostrea gigas*, to demonstrate stage-specific inbreeding depression during larval development (Plough and Hedgecock 2011) and the role of environmental stress in reducing the effects of genetic load (Plough 2012). Randomly bred wild-type lines of *C. gigas* can also experience significant mortality events, which may be attributed to naturally large genetic loads, resulting in high variation in specific combining ability, potentially as a function of extremely high mutation rates in wild populations (Plough et al. 2016). One proposal is that temporally balanced selection, evidenced by dynamic allele frequency changes in developing Pacific oyster larvae, could be an evolutionarily mechanism which simultaneously maintains genetic diversity in populations and improves average survival rates through a “shielding” of high genetic load during larval stages, from a larval population perspective (Durland et al. 2021; Durland et al. 2022). Studies with direct genetic measurements to address genetic load, loci correlated with survival and other commercially relevant phenotypes, and larval selection mechanisms in other bivalve species remain scarce.

The tri-species “*Mytilus* complex” consists of the blue mussel (*Mytilus edulis)*, the Bay mussel (*Mytilus trossulus*), and the Mediterranean mussel (*M. galloprovincialis*), each of which is capable of interbreeding and are intensively farmed in various locations throughout the world. The *Mytilus* complex represents 1 of the best scientific model systems for bivalves because of their commercial importance, a recently published genome (Murgarella et al. 2016), and many associated genetic and transcriptomic studies (Venier et al. 2003; Venier et al. 2009; Romero et al. 2011; Rosani et al. 2011; Suárez-Ulloa et al. 2013; Yusa et al. 2013; Lockwood et al. 2015; Paterno et al. 2019; Gerdol et al. 2020). *Mytilus* complex species have a rich literature history as a marine population genetics model to date, because of their commercial importance (Hilbish et al. 2012; Fraïsse et al. 2014; Coolen et al. 2020), because they can be considered invasive in certain habitats (Lins et al. 2021), and because of their outsized ecological role: they are sentinel species for marine pollution due to their sensitive larval stages and are ecosystem engineers (Borthagaray 2007; Wu et al. 2020). Despite increasing development of genomic resources for bivalves, especially for mytilids and the Pacific oyster in recent years, longitudinal genome-scale data remain scarce for 3 primary reasons: (1) bivalves have unique life histories, which present challenges to obtain time-scaled and granular genetic information, (2) early life stages are microscopic and difficult to assess without an experimental hatchery, and (3) selection pressures on marine bivalves are difficult to study as their natural ocean environment is constantly changing across spatial and temporal scales. The studies which have investigated short temporal scales in bivalves demonstrate that environmental stressors, such as increasing pH (Bitter et al. 2019; Durland et al. 2021), low salinity (McCarty et al. 2022), and temperature (Zhu et al. 2016), can affect bivalve population genetics over short windows of time and are especially acute for larval stages. Studies investigating wild adult populations of Pacific oysters, *C. gigas*, over long temporal periods (20 + years) suggest that random genetic drift, as a function of sweepstakes reproductive success and high larval dispersal capacity (Hedgecock and Pudovkin 2011), is responsible for long-term spatial genetic similarity, rather than natural selection for any particular genomic profile (Hedgecock and Pan 2021). However, Hedgecock and Pan (2021) also demonstrated that selective breeding can result in significant genetic divergence among selected lines compared to wild populations, and in the hatchery, genetic diversity has been preserved over generations of mass selections in the Pacific oyster based on microsatellite data (Hedgecock and Pudovkin 2011; Han et al. 2019; Hedgecock and Pan 2021; Chen et al. 2022). *Mytilus* complex species have been shown to exhibit geographic population structures at oceanic scales (Rio-Lavín et al. 2022) and hybridize at different rates at kilometer scales, indicating that mechanisms maintaining genomic stability and population structure must also be present in the complex. It is highly likely that environmental heterogeneity acts as a selection force to maintain a high level of genetic diversity in bivalves (Thomsen et al. 2017).

**Fig. 1.**
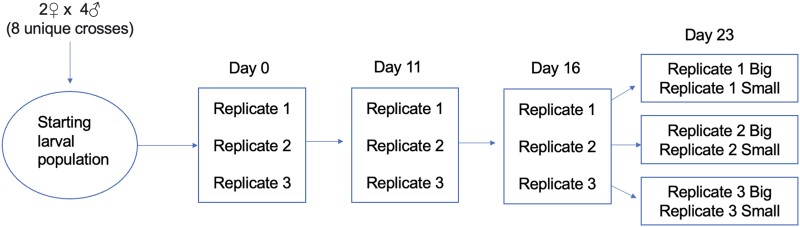
Experimental schematic. Cross design, replication, and sampling strategy throughout the experiment is shown. Family naming follows the format: *Fx.Mx.Rx.Dx*, where x, relevant number; F, female broodstock; M, male broodstock; R, replicate; and D, the day that the data w taken and size description, if necessary, e.g. big or small.

Here, we have designed a family breeding study to help develop a clearer understanding of the genetics and selection mechanisms experienced by larval cohorts of Mediterranean mussel in an attempt to inform commercial and scientific breeding interests for the mussel industry. We identify possible survival and size-associated loci for the Mediterranean mussel using a 2 female by 4 male crossing scheme (Fig. 1), followed by subsequent genomic analysis of segregating alleles among larval age cohorts in their progeny. Our analysis shows that allele frequencies change in a temporally dynamic way throughout several larval stages and suggests that balancing selection may maintain genetic variation in larval populations.

Recruitment in wild mussel populations has declined in the past 2 decades, in part as a function of intensive fishing and farming activities, resulting in difficulties in collecting wild mussel seed for aquacultural purposes and a trend toward hatchery-based mussel spat production (Pronker et al. 2008). Growers are considering the potential of harnessing GS for genetic improvement for *Mytilus* complex crops, and we anticipate that these viability and growth rate genomic data sets will be useful in the optimization of broodstock development and for informing efficient and functional breeding programs.

## Material and methods

### Broodstock source

Mediterranean mussels, *M. galloprovincialis*, were obtained via SCUBA diving the oil rig “Beta Offshore Platform Ellen,” in the Los Angeles channel (33.582968, −118.128579). Mussels were hand collected and checked for species and gravidity before collection. Collections took place in early February 2017 and utilized a broodstock collection permit held by Catalina Sea Ranch (no longer in business).

### Family generation and animal husbandry

Family line husbandry followed a generally accepted norm for bivalve hatcheries, outlined in Helm and Bourne (2004). A brief recounting follows. Ripe broodstock were induced to spawn using the “thermal shock” method in a set of group spawning tanks (transferring between 12°C and 25°C seawater baths in 30-min intervals). Our lab has had little historic success by individually isolating mussels and subsequently inducing spawning, as these bivalves seem to prefer conspecific gametes and/or chemical signals in the water to cue coordinated spawns, unless specifically conditioned to be ready to spawn in a separate system. This presents the problem of family line contamination, as bivalves will inherently siphon some sperm and/or eggs when in a group tank. To alleviate the possibility of family line contamination, we developed a gamete collection raceway (GCR), which consisted of a 1-m long by 10-cm tall PVC pipe cut lengthwise, with a water entry on an elevated side and a water exit on the downhill end, so as to produce a unidirectional water flow through a trough.

Individual mussels who were observed to begin the process of spawning in the group spawn tank were promptly isolated, washed, and allowed to continue spawning in the GCR for ∼10 min, thereby expelling any contaminated (i.e. fertilized in an uncontrolled manner) gametes inhaled. Individuals were then moved to isolated tanks to resume spawning for ∼1 h, at which point the gametes were collected and utilized for crosses. Tests of the GCR system reduced contamination to undetectable levels, as quantified by a complete lack of observations of fertilized and/or dividing embryos in collected egg clutches (data not shown) and confirmed by minimal loci which cannot be explained by heredity in this study. After collection of sperm or eggs, single parent crosses were generated by titrating sperm into an egg solution until ∼10 sperm could be observed surrounding each egg on a subsample slide. After ∼30 min, the eggs were washed with 1 *µ*m filtered seawater (hereafter FSW) to avoid polyspermy effects. After fertilization, embryos were quantified using a Sedgewick rafter and associated recommended counting protocol (see Helm and Bourne 2004) and divided into 12L tanks at an even starting density of 10,000 fertilized embryos per liter. Starting family size was therefore 120,000 individuals per replicate. Note that the coefficient of variation for embryo counts, calculated as the standard deviation divided by the average, was targeted to be between 0.1 and 0.2 (or lower), which ensured confidence in our counts for all populations mentioned. Families were raised in a family line tank system designed and created by Nathan Churches at the Wrigley Institute for Environment Science (WIES) on Catalina Island in a temperature controlled space (Supplementary Fig. 2). The system allowed for consistent and even distribution of microalgal feed and aeration between replicate tanks, and the design was such that newly filtered FSW was constantly delivered from the WIES intake into a 200L sump, which was then distributed to each of the individual replicate tanks. The water would overflow from each individual tank into a drainage system, and animals were kept in-tank using banjo filters. During feeding, the system would be removed from the WIES FSW intake and set to recirculation, after which microalgae feed was delivered to the sump and distributed to each individual tank, such that each tank received exactly the same amount of microalgae. After feeding was complete, the system was again returned to flow-through state, and new FSW was allowed to clear the system of food and detritus. Aeration was visually checked for similarity between tanks, and FSW delivery was kept consistent between tanks by use of agricultural irrigators (2 gph). Feeding schedules followed a target of 1 feeding episode per 3 days, with a density of ∼100,000 cells of *Isochrysis galbana* per milliliter, or equivalent nutritional count as determined by utilizing cross-species microalgae tables provided in Helm and Bourne (2004), for *Tetraselmis* spp. and *Chaetoceros* spp.; multispecies microalgae feed schedules were utilized after the larvae reached an appropriate size (∼100 *µ*m). Families were reared and phenotyped as described below and at day 80 settled onto 1-m length of “fuzzy rope,” eventually being outplanted on day 114 on a “commercial mock longline” in Cat Harbor, but unfortunately were lost due to access issues experienced during the COVID pandemic.

### Family design and phenotyping scheme

The mating design for this study was a full factorial cross (NC-II design) using 4 females and 2 males, with 3 tank replicates for each family, for a total of 8 families replicated across 24 tanks (Fig. 1). Each replicate was standardized in terms of stocking density, feed allocation and feed type, and water parameters tested (pH, temperature, and salinity), using an Onset Hobo thermocoupler K-type device and handheld YSI probe device. The larval stages of the family lines underwent a phenotyping protocol for survival and size on days 0, 11, 16, and 23. On day 23, the populations were further divided into 2 phenotypic ranges, large and small, using a tiered sieve system for size gradation (i.e. large mussel larvae were caught in 150 *µ*m sieves and isolated and small mussel larvae passed through the 150 *µ*m sieve and isolated). Size was measured using an Olympus SZ-PT dissecting microscope with Techniquip 150W Fiber Optic Illuminator, at 20× total magnification, and images were taken and analyzed using built-in DP2-BSW XV Imaging Processing Software. Counting for population survival was performed by capturing and concentrating the entire cohort by use of an appropriately sized mesh sieve and placing this concentrate into 50 mL conical vials. A small bubbler was place at the bottom of the conical vial, such that the cohort concentrate could be homogenized throughout the water column. At this point, several aliquots were retrieved and placed on a Sedgwick Rafter counting cell, 5% ethanol was added to each sample to reduce swimming capacity of larvae, and each aliquot was then quantified using a compound microscope at 4× magnification. All spawn-log excel charts and images are available upon request.

Samples were collected on each of the phenotyping days for population genomic sequencing (*n* individuals ranged from 48 to 11,625, depending on the population and date), including for both size cohorts (big and small) collected on day 23. The end result, after replication of families and division of sizes, was a total of 73 larval populations. Family naming follows the format *Fx.Mx.Rx.Dx*, where x, relevant number; F, female broodstock; M, male broodstock; R, replicate; and D, the day that the data were taken and size description if necessary. For example, the larval cohort sampled for genomic analysis on day 23 which was from the big population, and originating from female 1 and male 1, replicate 2, would be called *F1.M1.R2.D23Big.* Note that day 0 does not have replicates indicated, as genomic samples were taken from the fertilized population before delivery to replicate tanks.

### DNA extraction

DNA extraction protocols specific for bivalve DNA can be found in the supplemental documents section, Supplementary File 1. Briefly, for both adult and larval samples, a tissue digestion was performed using a diluted solution of pure proteinase K in Tissue and Cell Lysis buffer from Thermo Fisher Scientific (1:15 ratio). After appropriate tissue digestion, an MPC Protein Precipitation Solution was used to remove excess lipids and proteins, and a column extraction using a Zymo Research Quick-DNA Microprep kit was performed, followed by a column cleaning using a Zymo Research DNA Clean and Concentrate (DCC) kit. The extracted DNA was tested for molecular weight using a 1% agarose gel and tested for salt and other contaminants using a Thermo Scientific NanoDrop machine. DNA concentrations (and all other concentrations required throughout NGS library generation) were quantified using a Qubit 2.0 Fluorometer from Thermo Scientifc.

### NGS library generation

The full protocol for library preparation can found in Supplementary File 1. In brief, a 2-enzyme strategy was developed after determining that SphI-HF and Mlu-CI had adequate cutting behavior for the *Mytilus* genome, targeting fragments between ∼100 and 900 bp, as determined by a multienzyme assay (data not shown). Libraries were generated using a protocol adapted from Ithnin et al. (2021). Libraries were pooled, tested for quality control, and sequenced across 2 Illumina “NovoSeqS4” lanes by the Novogene Corporation.

### Data filtering

The number of reads that cover each marker for each family was plotted in a line histogram (e.g. Supplementary Fig. 1a, only 1 family is shown, all family line histograms available upon request). Minimum marker coverage for parents was filtered at 40× from common logarithms of read depth (Supplementary Fig. 1b). Initial analysis of these results confirmed that parental read depth in 7 replicates in 5 families did not meet the minimum threshold and was subsequently discarded. In addition, 2 families had insufficient data at day 0, leaving 8 replicates in 5 families for subsequent analysis.

For the remaining replicates which passed this initial filtration, read depth at all time points is at least 20× at all loci analyzed, meaning the number of observed counts for both reference and alternative alleles is at least 20× for each marker at all times. For markers that have allele counts which have recovered from a 0 at 1 time point and subsequently reestablished at the next time point (a biological impossibility), their allele counts at the 0 time point have been converted to missing data.

### Allele frequency trend during experimental time

Allele frequencies for all markers in each replicate were calculated using equations 2 and 3, below. For each time point and size group, the average allele frequency was calculated as the mean of all markers. Additional details and script of allele frequency trend can be found in Supplementary Table 1; see also the *Data availability* section below.

### Pearson's chi-square test for heterozygous markers between day 0 and day 23

A chi-square test of independence was used to determine whether there was a significant difference in the reference and alternative alleles read counts between day 0 and day 23. A 2 × 2 contingency table was generated for every heterozygous marker, with row variables being the reference and alternative allele reads and column variables being days. Chi-square tests for significance were carried out for all replicates across families, with significance reported using equation 1:


$$\alpha =\frac{0.05}{nHet}$$

Where ***α*** is the significance threshold, and ***nHet*** is the total number of heterozygous markers in the replicate. This is an alternative to the conservative Bonferroni correction and is more rigorous than an FDR approach. For replicates that have data for both big and small phenotypic groups on day 23, 2 contingency chi-square tests were performed for every marker, 1 between day 0 and day 23 big (hereby referred to as D23Big) and the other between day 0 and day 23 small (hereby referred to as D23Small). Markers that met the significance threshold or both tests were reported. Detailed and completed *P*-values for all markers can be found in Supplementary Table 2.

### Pearson's chi-square test for allele counts supporting female and male haplotype between day 0 and day 23

Reference and alternative allele frequencies at each time point were calculated based on read counts. Reference allele frequency (***ref-AF***) was calculated using equation 2:


$$ref-AF=\frac{\;r.rc\;}{s.arc\;}$$

where ***r.rc*** equals the reference allele read counts at the locus and ***s.arc*** equals the sum of all read counts (reference and alternative). Alternative allele frequency (***alt-AF***) was similarly calculated using equation 3:


$$alt-AF=\frac{a.rc\;}{s.arc}$$

where ***a.rc*** equals the alternative allele read counts.

The parental genotypes at all analyzed markers were required to return homozygous within the individual parent and heterozygous between them, making the marker in the subsequent larval F1 generation heterozygous and allowing for tracking of maternal and paternal alleles. Allele read counts on day 0 and day 23 were used to calculate female haplotype and male haplotype. A chi-square test was used to determine whether there was a significant difference in the female and male alleles read counts between day 0 and day 23. *P*-values for each marker were converted to *q*-value in the R package *q*-*value*. For replicates which have data for both big and small phenotypic groups on day 23, 2 chi-square tests were performed for every marker. The *P*-value and *q*-value for all markers can be found in Supplementary Table 3. Markers that met the significance threshold of *q*-value < 0.05 for both tests were reported and can be found in Supplementary Table 4.

### Contig analysis

Contig analyses were done based on parental haplotypes. The total allele counts supporting the parental haplotypes were calculated as the sum of allele reads supporting parental genotypes at every marker present in the contigs. Thus, there were 2 total allele counts reported for every contig, 1 based on female haplotype and the other based on male haplotype. A chi-square test was used to determine whether there was a significant difference in the female and male contig read counts between day 0 and day 23. A 2 × 2 contingency table was set up for every contig, row variables being female and male contig reads and column variables being days. A *P*-value and a *q*-value were reported for all contigs. *P*-values for each contig were converted to *q*-value in the R package *q value*. A *P*-value and a *q*-value were reported for all contigs and can be found in Supplementary Table 5. Contigs that met the significance threshold of *q*-value < 0.05 were reported. For replicates that have data for both big and small phenotypic groups on day 23, 2 chi-square tests were performed. Contigs that met the significance threshold for both tests were reported and can be found in Supplementary Table 4. The number of significant contigs reported can be found in Supplementary Table 6.

A chi-square test was used to determine whether there was a significant difference in the female and male contig read counts between day 16 and day 23. For day 23, data for big phenotypic groups were used in the chi-square tests. A *P*-value and a *q*-value were reported for all contigs. The overlap among contigs that met significance thresholds from both contig analyses are reported in the final results. Detailed information about the overlapped significant contigs can be found in Supplementary Table 7.

### Selecting markers with consistent allele frequencies change

Allele frequencies were calculated using equations 2 and 3, above. Allele frequencies at day 11, day 16, day 23Big, and day 23Small were normalized to day 0. From the contigs identified in the previous analyses, markers with consistently increasing or decreasing allele frequencies were reported (Supplementary Table 8).

### Pearson's chi-square test for heterozygous markers on day 23 between the 2 phenotypic groups

A chi-square test of independence was used to determine whether there was a significant difference in the reference and alternative alleles read counts on day 23 between the 2 phenotypic groups, big and small. A 2 × 2 contingency table was set up for every heterozygous marker, row variables being the reference and alternative allele reads and column variables being groups. Chi-square tests for significance were carried out for all replicates across families, with significance reported at the adjusted significance level (α). Markers that met the significance threshold can be found in Supplementary Table 9.

## Results

The mating design for this study was a full factorial cross (NC-II design) using 4 females and 2 males, with 3 grow out tank replicates for each family, for a total of 8 families replicated across 24 tanks (Fig. 1). On days 0, 11, 16, and 23, samples were collected for population genomic sequencing and underwent a phenotyping protocol for survival and size. On day 23, the populations were further divided into 2 phenotypic ranges, big and small, based on their ability to pass through a 150 *µ*m sieve. A bioinformatics pipeline was established from the pooled-DNA sequencing data to select viability and size-associated SNPs.

### System conditions controls

In order to ensure that variance in abiotic conditions between tanks was nominal, we took temperature, pH, salinity, and percent dissolved oxygen measurements in the hatchery system described above. The system was kept on FSW flow-through for the majority of the rearing process, and system samples of pH and salinity remained consistent across time (pH, average 7.96; SD 0.013; salinity, 31.99 parts per thousand; SD 0.991), and between-tank temperatures were taken every 30 s for a 22 day period (2017 February 17 to 2017 March 10) and demonstrated minimal differences (typically <1°C or less) (Supplementary Fig. 2). This was consistent with several preliminary results indicating that temperature was stable between tanks in our system (data not shown). Flow was adjusted to the system such that no tank would risk clogging or over flowing, and we estimated that the system (∼500 l) replenished itself approximately twice each day when on the flow-through setting.

### Library generation

ddRAD libraries generated and sequenced across 2 lanes of NovoSeqS4 Illumina returned with a total of 5.65B and 6.13B reads, respectively, for a total data set of ∼11B reads identifiable to individual after filtering. Three samples, F1.M4.D0, F1.M2.D0, and F2.M1.R1.D23 returned only 2,626, 201,888, and 2.26 M known reads, respectively, while 1 sample (F1.M4.R2.D11) exceeded the average significantly with 107 M reads. Excluding these outliers, the range was 89.1 M, with a maximum of 93.9 M reads (F2.M2.R2.D11) and a minimum of 4.8 M reads (F1.M4.R3.D23Small). Including all sample except F1.M4.D0, the data set has an average depth of 53 and 56 M per sample for columns 1 and 2, respectively. The total data set consists of ∼11B known reads. A general display of reads can be seen in Supplementary Fig. 3, with heat mapping to indicate extreme outliers. A total of 8 family replicates report complete congruence between presence of a genomic sample and phenotypic profile for growth and survival, while another 5 cohorts have congruence through day 23 (D23) but have only a big (D23Big) population recorded on that day. One family replicate (F2.M4.R1) has all but D16 recorded for genomic profiles. Family F1.M4 was the only family to have phenotyping and genomic data across all possible time points and replicates, while all other families have some portion of data missing through at least 1 replicate. The above is summarized in Supplementary Fig. 3b for column 1. Read count for column 1 and column 2 is highly similar (Supplementary Fig. 3c).

### Survival and growth

**Fig. 2.**
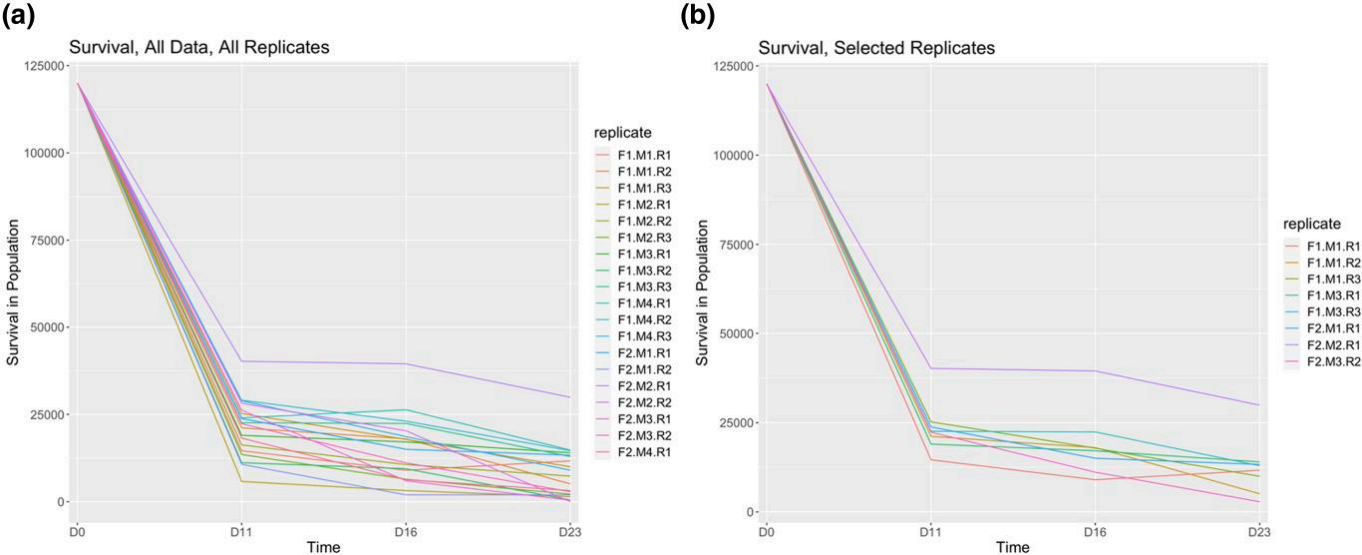
Larval survival from day 0 to day 23 for all experimentally produced families and replicates. a) This graph demonstrates survival for all replicates across all time points and illustrates the high frequency of type III survival in the Mediterranean mussel. b) This figure demonstrates survival for the 8 selected replicates after data filtering across all time points.

**Fig. 3.**
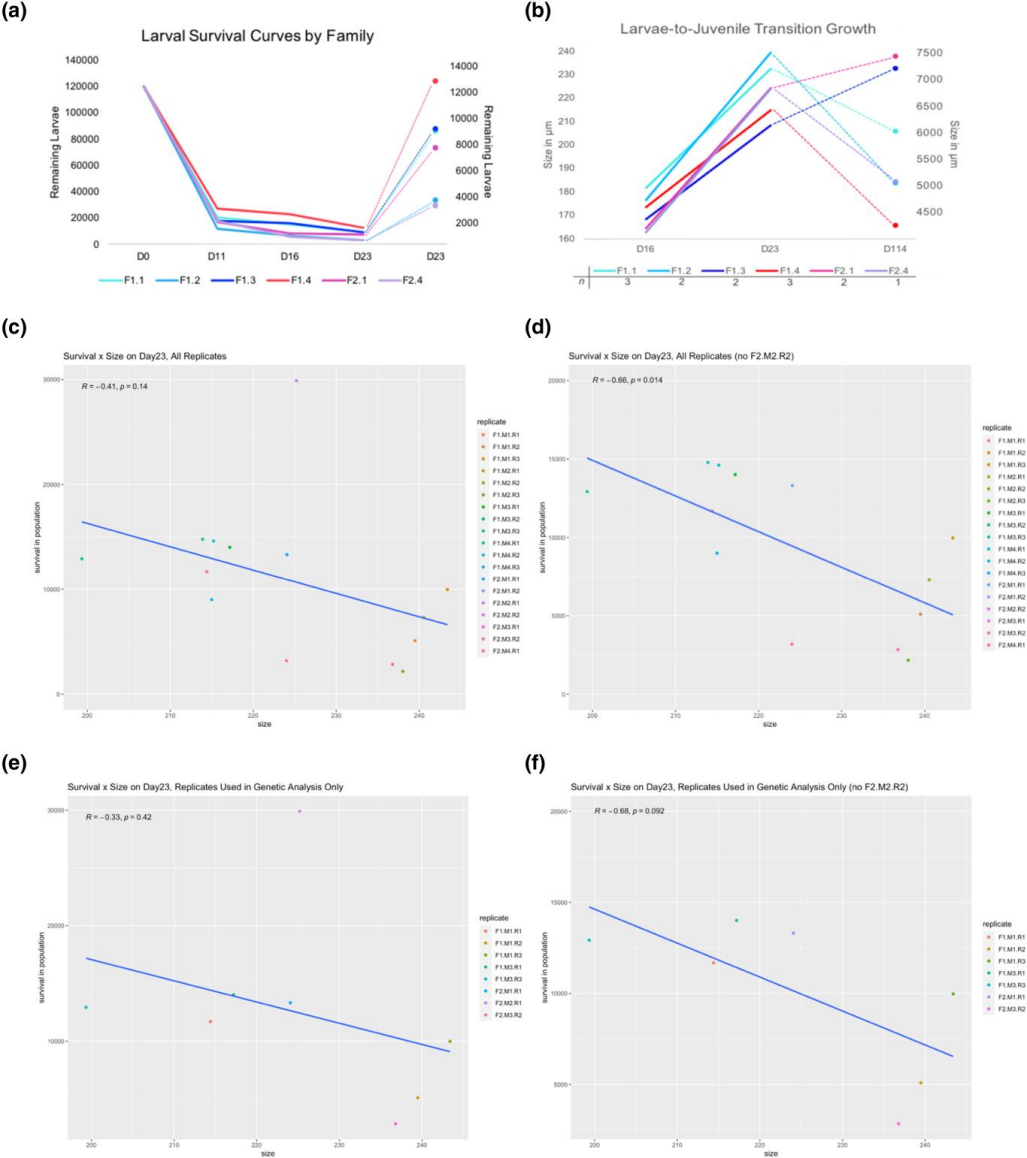
Larval size and survival correlations for full data set. a) Larval survival curves are shown, averaged per family for day 0 (D0) through day 23 (D23). The secondary *y*-axis on the right is scaled down for better visualization of the data. b) Growth rates are shown between day 16 (D16) and day 114 (D114), just before outplanting at an open environment site. c) Correlations between average size per family and survival at day 23 (D23) are shown in all replicates. d) Correlations between average size per family and survival at day 23 (D23) are shown, all replicates without outlier replicate F2.M2.R1. e) Correlation between average size per family and survival at day 23 (D23) in selected replicates after data filtering. f) Correlation between average size per family and survival at day 23 (D23) in selected replicates after data filtering without outlier replicate F2.M2.R1. Each individual replicate is separated; a linear regression and associate *R*-values are shown.

Survival curves was observed to be semi-unstable in terms of consistency of order among all cohort replicates across time (Fig. 2), but a stabilization of the hierarchy among families seems to have occurred somewhere around settlement (Fig. 3a, day 16 to day 23 transition). Growth rates differed between families significantly at various time points. For example, an ANOVA and subsequent Tukey's test from data taken on day 16 indicate that certain cohorts may be performing, on average, better than others both within family (e.g. F1.M1.R1.D16 vs F1.M1.R3.D16) and among families and replicates (Supplementary Fig. 4). However, the general trend was not predictive through multiple time points (Supplementary Fig. 4d) or to the settlement stage and outplanting stage (day 114; see Supplementary Fig. 4c). There was a likely significant negative correlation between survival and size for all replicates once 1 outlier replicate was removed (Fig. 3d; R: −0.66), with significance threshold reported at α = 0.05 for a Pearson's test. There seemed to be similar negative correlation between survival and size for the selected replicates after data filtering, but it did not meet the significance threshold (Fig. 3f).

### Selection of viability-associated SNPs

With replicated cultures and a pooled sequencing approach, we selected viability-associated SNPs based on 2 measurements. We first identified SNPs that changed significantly in allele frequencies between the larvae sampled on day 0 and the day 23 larval population in each replicate. Significant SNPs were identified using Pearson's chi-square tests to compare start-point allele counts to end-point survivor counts, hereafter referred to as viability-associated SNPs (Table 1, columns 4–8, and Supplementary Table 2). We detected 192, 4,342, and 4,055 loci in 3 replicates from family F1.M1, all of which were above the set significance level of:


$$\;\alpha =\frac{0.05}{n\mathrm{Het}}$$

**Table 1.** Potential viability-associated SNPs and size-associated SNPs.

Family	Replicate	Total # of markers	D0 vs D23Big	D0 vs D23Small	Viability-associated SNPs	Overlap SNPs	% of viability-associated SNPs	Increasing pooled allele frequency from D0 to D23	Decreasing pooled allele frequency from D0 to D23	% of viability-associated SNPs	Size-associated SNPs	% of size-associated SNPs
Female 1 x male 1	1	5516	1269	404	192	149	3.481%	17	13	0.54%	320	7.23%
	2	23741	4342	NA	4342		18.29%	218	200	1.76%	NA	NA
	3	24,299	4055	NA	4055		16.69%	238	242	1.98%	NA	NA
Female 1 x male 3	1	2626	234	78	23	10	0.876%	1	0	0.04%	88	4.58%
	3	8564	759	305	124		1.448%	4	12	0.19%	306	4.58%
Female 2 x male 1	1	1101	87	73	18	NA	1.635%	3	6	0.82%	46	6.29%
Female 2 x male 2	1	3590	393	247	75	NA	2.090%	11	8	0.53%	181	7.09%
Female 2 x male 3	2	21,337	1192	*NA*	1192	NA	5.59%	167	159	1.53%	NA	NA

Total number of markers in column 3 indicates total number of heterozygous markers in each replicate after data filtering. Columns 4–8 are viability-associated SNPs with significant changes in allele frequencies on day 0 (D0) and day 23 (D23). The number of potential viability-associated SNPs which were significant from chi-square tests for both big and small phenotypic groups, is shown in column 6. The significance threshold is set at α = (0.05/total number of heterozygous markers in the replicate). D0 vs D23Big in column 4 indicates number of SNPs with significant changes in allele frequencies on day 0 and day 23Big from chi-square tests. D0 vs D23Small in column 5 indicates number of SNPs with significant changes in allele frequencies on day 0 and day 23Small from chi-square test. Percent of markers in column 8 is calculated as viability-associated SNPs (column 6)/total # of markers (column 3). Columns 9–11 are potential viability-associated SNPs with consistent changes in allele frequencies from day 0 to day 23. “Increasing” indicates viability-associated SNPs with allele frequency increasing from day 0 to day 23; “decreasing” indicates viability-associated SNPs with allele frequency decreasing from day 0 to day 23. Percent of markers in column 11 is calculated as viability-associated SNPs (sum of column 9 and column 10)/total # of markers (column 3). Columns 12–13 are size-associated SNPs with significant changes in allele frequencies on day 23 between the 2 phenotypic groups (big vs small). Percentage is calculated as size-associated SNPs (column 12)/total number of markers (column 3).

where **α** is the significance threshold, and **nHet** is the total number of heterozygous markers in each replicate. Statistically significant changes in allele frequency at the adjusted level (**α**) range from 0.87 to 18.29% of markers in the 8 replicate cultures. The numbers of significant markers vary among the 3 replicates within the same family because the number of heterozygous markers differ largely in the replicates. F1.M1.R1 has 5,561 heterozygous markers, but F1.M1.R2 and F1.M1.R3 have more than 20,000 heterozygous markers. To identify a list of significant SNPs for family F1.M1, we compared all chosen SNPs in the 3 replicates to find common loci. This resulted in the identification of 149 overlapping SNPs in family F1.M1. These shared SNPs are important as these replicates in family F1.M1 capture random sources of variation and thus limit the impact of errors and noise in our analysis. There are 10 shared SNPs from the 2 replicates (R1 and R3) of family F1.M3. None of the other families have more than 1 replicate in which to find common loci.

We next performed contig analysis in order to find contigs that had the greatest genetic changes. Supplementary Tables 5–7 in the supplemental documents contain a list of all overlapping contigs in each replicate and a complete list of significant contigs. From the complete list of contigs, we identified SNPs with consistent changes in allele frequencies across replicated cultures from day 0 to day 23 (Table 1, columns 9–11, and Supplementary Table 8). Allele frequencies which were uniform in direction (either consistently increasing or consistently decreasing) at each of the 4 time points were considered viability loci. We detected 30, 418, and 480 loci in 3 replicates from family F1.M1, respectively (Table 1, columns 9–10); the variance in viability loci between replicates is a function of the total number of heterozygous markers identified in each replicate. However, there was no overlap between loci or contigs among the 3 replicates in family F1.M1. The absence of shared viability loci among replicates within the same family could result from either (1) a disparity in the total number of starting markers in the 3 replicates, or (2) our stringent significance selection criteria were overly sensitive to false positives. This approach was more rigorous than the chi-square tests in our previous analysis, as it took all 4 time points into consideration. Here, changes in allele frequencies were related to a shift in parental genotype composition.

### Selection of size-associated SNPs

Size separation on day 23 divided larvae into 2 groups in 5 replicates across 4 families. Hereafter, these groups will be referred to as the big groups (Big) and small groups (Small), respectively. We identified SNPs that changed significantly in allele frequencies between the 2 phenotypic groups sampled on day 23 in each replicate, hereafter referred to as size-associated SNPs (Supplementary Table 9). The number of size-associated SNPs is reported in Table 3 and ranges from 46 to 320 identified SNPs. The percentage of size-associated SNPs (compared to all SNPs identified) in each replicate was comparable, ranging from 4.58 to 7.23% (Table 1, column 13). Size-associated SNPs were then compared to the sets of viability loci from our contig analysis, resulting in only 1 overlapping site in F1.M1.R1, while comparison of size-associated SNPs to viability-associated SNPs from chi-squared tests resulted in a range of overlapping sites depending on the replicate, from 0 to 27 (Table 2 and Supplementary Table 10). Overlapping sites in these analyses have a high potential for contributing to both size and survival, each commercially relevant phenotypes.

**Table 2.** Overlap between size-associated SNPs (Table 1, column 12) and survival-associated SNPs.

Family	Replicate	Size-associated SNPs	Viability-associated SNPs (chi.sq test)		Viability-associated SNPs (Consistent increasing/decreasing trend)	
			Total number	Overlap with size-associated SNPs	Total number	Overlap with size-associated SNPs
Female 1 × male 1	Replicate 1	320	192	27	30	1
female 1 × male 3	Replicate 1	88	23	0	1	0
	Replicate 3	306	124	17	16	0
Female 2 × male 1	Replicate 1	46	18	2	9	0
Female 2 × male 2	Replicate 1	181	75	3	19	0

Number of survival-associated SNPs from chi-square test (Table 1, column 6) is indicated in column 4. Number of overlapped SNPs between size-associated SNPs (Table 1, column 12) and survival-associated SNPs (Table 1, column 6) is shown in column 5. Number of survival-associated SNPs from uniform changes (Table 1, columns 9–10) is indicated in column 6. Number of overlapped SNPs between size-associated SNPs (Table 1, column 12) and survival-associated SNPs (Table 1, columns 9–10) is shown in column 7.

**Table 3.** Sequence divergence and heterozygosity of viability-associated SNPs from chi-square test.

Family	Replicate	Total number	Day 0	Day 11	Day 16	Day 23B			Day 23SM		
			Heterozygosity	Heterozygosity	Heterozygosity	Increase sequence divergence	Decrease sequence divergence	Heterozygosity	Increase sequence divergence	Decrease Sequence Divergence	Heterozygosity
Female 1 × male 1	Replicate 1	192	0.1553421	0.2125114	0.2009738	97 (50.52%)	95 (49.48%)	0.2102884	90 (46.88%)	102 (53.13%)	0.1892238
	Replicate 2	4342	0.1968872	0.2192123	0.2162645	2,061 (47.46%)	2281 (52.53%)	0.2093594	NA	NA	*NA*
	Replicate 3	4055	0.1955834	0.2188703	0.2147474	1,927 (47.52%)	2128 (52.48%)	0.2108777	NA	NA	*NA*
Female 1 × male 3	Replicate 1	23	0.1075425	0.1794699	0.2022685	7 (30.43%)	16 (69.57%)	0.2004655	7 (30.43%)	16 (69.57%)	0.1675017
	Replicate 3	124	0.1045893	0.2011598	0.2073399	45 (36.29%)	79 (63.71%)	0.2197237	39 (31.25%)	85 (68.54%)	0.1846072
Female 2 × male 1	Replicate 1	18	0.1101242	0.2208561	0.2086362	5 (27.78%)	13 (72.22%)	0.2100069	5 (27.78%)	13 (72.22%)	0.1835012
Female 2 × male 2	Replicate 1	75	0.1494119	0.2106503	0.2187494	35 (46.67%)	40 (53.33%)	0.2257203	34 (45.33%)	41 (54.67%)	0.1675987
Female 2 × male 3	Replicate 2	1192	0.1975513	0.2105287	0.2098813	562 (47.15%)	630 (52.85%)	0.1731810	NA	NA	*NA*

Total number in column 3 is the number of viability-associated SNPs from chi-square tests (Table 1, column 6). Heterozygosity at each locus is calculated as the multiplication of reference allele frequency and alternative allele frequency. There is a slight increase in heterozygosity from day 0 to day 23. On day 23, increase sequence divergence means an increase in alternative allele frequency. Decrease sequence divergence means a decrease in allele frequency in the alternative allele, thus an increase in reference allele frequencies.

## Discussion

**Fig. 4.**
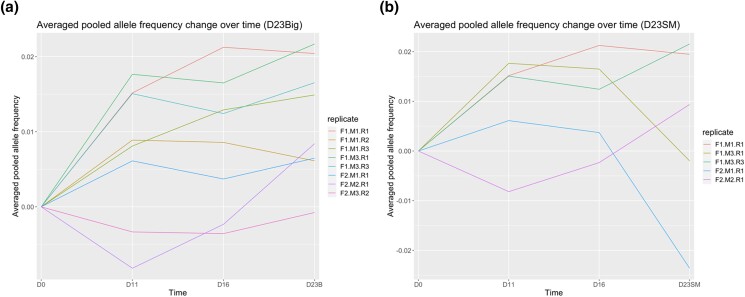
Average pooled alternative allele frequency change across replicates over 23 days of early *Mytilus galloprovincialis* development in big vs small larval populations. On day 23, the allele frequency trends are separated by size group. a) B indicates big phenotypic groups. b) SM indicates small phenotypic groups. Allele frequency in this figure was calculated as (alternative allele read counts)/(reference allele read counts + alternative allele read counts). For each time point, average alternative allele frequency was calculated as the mean of all markers.

We used changes in allele frequency in larval populations (day 0 to day 23) of the Mediterranean mussel, *M. galloprovincialis*, to identify potential viability-associated SNPs. Using chi-squared tests, we identified thousands of SNPs that were correlated with viability across several families and replicates during the larval development period (Table 1, column 6, and Supplementary Table 2, Supplementary Table 10). However, the majority of alleles showed patterns of changes in allele frequencies which were inconsistent with simple explanations of genetic purging (inbreeding-mediated negative selection against deleterious alleles) or directional selection. Our analysis indicates that balancing selection may be the mechanism maintaining genetic variation in the larval population, because the majority of allele frequencies changed in a temporally dynamic way throughout the course of the experiment (Fig. 4). Balancing selection is usually utilized as an umbrella term encompassing processes, whereby genetic variation is adaptively preserved in a population at a greater frequency than can be explained from genetic drift (Stahl et al. 1999; Bergland et al. 2014; Llaurens et al. 2017). Balancing selection is evidenced by an excess of intermediate frequency variants and by regions of increased polymorphism around a selected site (Siewert and Benjamin 2017). This is 1 of the primary mechanisms in which genetic variation can arise and has been demonstrated in most major taxa, for example, in plants (Weinig et al. 2003) and oysters (Durland et al. 2021; Durland et al. 2022). Genetic variation at functional loci across the genome is likely retained via multilocus balancing selection within populations experiencing temporally fluctuating selection pressures (Bergland et al. 2014; Brennan et al. 2019). There has been substantial evidence of the maintenance of genetic diversity by temporal balancing selection in many organisms, such as oysters (Durland et al. 2021; Durland et al. 2022), *Drosophila* (Chakraborty and Fry 2016; Chapman et al. 2019), and even humans (de Bakker et al. 2006; Andres et al. 2009). Several processes are thought to contribute to balancing selection, including heterogeneous selection, where heterozygous individuals have a fitness advantage over either homozygote (Chapman et al. 2009). Among the viability-associated SNPs in our data, none appeared to be uniformly advantageous or disadvantageous for either reference or alternative alleles (i.e. no maternal or paternal selection, Table 3), and we did observe a slight increase in heterozygosity in most replicate cultures on day 23 when compared to day 0 (Table 3). This observed pattern does not fit well with directional selection models, whereby advantageous, viability-associated alleles are selected for across multiple larval time points which results in a loss of overall genetic diversity due to linkage disequilibrium. Rather, our observations could be explained by improved fitness in the heterozygous state, whereby no single allele is beneficial across all larval time points in the majority of cases.

SNPs which showed consistent changes in allele frequencies across replicate cultures throughout the duration of the experiment could also be attributable to selection for genetic variation that impacts survival (viability-associated SNPs; Table 1, columns 9–10, Supplementary Table 8). Under a model of unlinked drift, approximately half of all loci would show changes in allele frequency between 2 time points, but it would be unlikely that a locus has a consistent shift in allele frequency over all 4 time points by chance (drift) alone. We modeled the potential for our results to be explained by drift alone by calculating starting allele frequencies at all 4 time points and found an average of 12.3% (SD = 0.67) chance-of-drift for all viability-associated SNPs (see Supplementary Table 11). While random genetic drift may have caused changes in allele frequencies at some loci (Supplementary Table 11), it is mathematically very unlikely (∼0.123^8^) that drift is the causative factor for *all* of viability-associated SNPs identified in Table 1, columns 9–10. We also argue that it is improbable that drift alone is the causative factor in all viability-associated SNPs for 2 logical reasons. First, the literature suggests that bivalves exhibit on the order of tens-to-dozens of viability-associated sites per generation (Plough et al. 2016; Yin and Hedgecock 2021), putting our estimates within or nearly within the same order of magnitude as current studies. Second, the proportion of SNPs associated with viability compared to total markers analyzed is quite small (0.04 to 1.76%, Supplementary Table 11), which indicates that balancing selection is the primary selective mechanism at the majority of genomic loci and that any outliers (e.g. viability-associated SNPs) must overcome this pressure and are likely to be related to real phenomenon, as opposed to drift alone. Future work will need to confirm these results by genetic manipulation or selective breeding studies.

Oligogenic models of inheritance, whereby a handful of distinct genes or loci are responsible for large effects, is a possible mechanism which may explain the relatively small number of loci involved with either viability or size in this analysis, but studies describing the mechanistic basis of larval growth and development are rare, making oligogenic arguments difficult. Growth rates and survival rates in bivalves are governed by both environmental and biological factors. Exogenous factors, such as temperature and food intake (Wang et al. 2010; Joubert et al. 2014), have been shown to have an influence on bivalve growth performance (total body weight, shell length, shell height and shell width, etc.). However, the biological factors behind these traits are still poorly understood. Although little comparable genome-wide data are available for any bivalve species during larval development, transcriptomic studies have been done to identify important development-related genes (Clark et al. 2010; Meyer and Manahan 2010). Recent studies have suggested that heterosis is the causal factor behind differential growth rates of bred family lines and can be experimentally produced in early development of the Pacific oyster *C. gigas* (Pace et al. 2006; Hedgecock et al. 2007). Several genes have been identified as potential growth candidates as they are associated with processes such as energy metabolism and protein metabolism (Meyer and Manahan 2010).

The variety of different allele frequency change patterns is representative of an underlying polygenic condition for larval survival and growth in wild Mediterranean mussels, consistent with an emerging body of work indicating that survival can exhibit a polygenic basis (Hilbish et al. 2012; Fraïsse et al. 2014; Lins et al. 2021). Previous transcriptomic analyses of other bivalves demonstrate that these species have complex developmental processes, and selective pressures taking place in the early stages are very unlikely to be uniform across their entire life cycle due to the dynamic oceanic environment in which they live (Rodríguez-Trelles et al. 2013; Campbell-Staton et al. 2017; Brennan et al. 2019). Thousands of genes have been shown to have stage-specific expression throughout the development of several species (Cruickshank and Wade 2008; Heyland et al. 2011; Huan et al. 2012; Song et al. 2016), including bivalves (Plough 2018). Maintenance of high levels of heterozygosity could be explained by stage-specific gene expression, as multiple alleles could be responsible for different effects at different stages of development, or each allele may be associated with 1 or more interacting genes which themselves exhibit temporal fluctuations in fitness. In other words, an advantageous genotype during 1 developmental stage may lose its beneficial effects later, which could lead to changes in gene expression patterns, and these shifts may occur over short temporal timescales (hours to days). Similar stage-specific gene expression has been observed and studied in *C. elegans* (Hendriks et al. 2014; Laxman et al. 2014) and *Drosophila* astrocytes (Huang et al. 2015). Another possible explanation, as well as 1 which is not mutually exclusive to the stage-specific selective pressures already mentioned, is that these SNPs are linked to 2 or more genes that are under repulsive selective pressures. For example, in the Pacific oyster *C. gigas*, genetic marker Cg205 was shown to be linked to 2 different recessive mutations in repulsive phase, each with different patterns of selection during metamorphosis (Plough and Hedgecock 2011; Plough 2018). Observed fluctuations in allele frequencies in this study may reflect stage-specific genetic changes that are temporally balanced across the early developmental period for Mediterranean mussels.

**Fig. 5.**
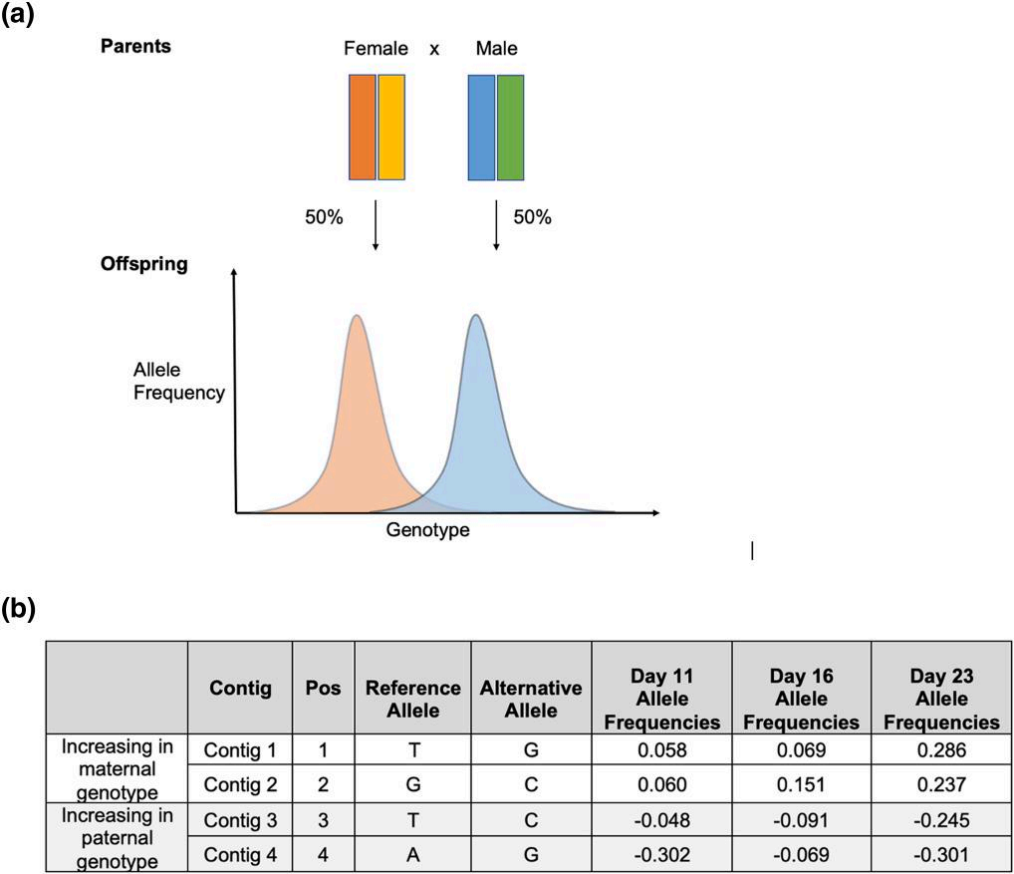
Average pooled allele frequencies supporting parental haplotypes in F1 *Mytilus galloprovincialis* larval populations. a) Experimental design. Female has two chromosomes represented by the two bars on the left (maternal genotype), male has two chromosomes represented by the two bars on the right (paternal genotype). b) Illustration of allele frequency changes. Day 11 (D11), day 16 (D16), and day 23 (D23) are alternative allele frequencies normalized to alternative allele frequencies on day 0 (D0).

We found a rather consistent number of SNPs exhibiting significant allele frequency changes on the starting time point (day 0) and endpoint (day 23) (Table 3) and a consistent number of SNPs having an increasing or decreasing allele frequency across all time points (Table 1, columns 9–10). This pattern of balanced changes in allele frequencies appeared to occur in all replicates. These bidirectional allele frequency trajectories are in accordance with our experimental design (Fig. 5a). All analyzed markers were filtered to be heterozygous for day 0 larval populations, meaning that each parent passed down 1 of its genetic variants to the offspring. Given this, we expected to see a uniform distribution of parental genotypes in the offspring. Figure 5b is a simplified illustration of balanced bidirectional changes for 1 example contig. For all heterozygous markers in the F1 generation, their reference alleles come from the maternal haplotype, and the alternative alleles come from the paternal haplotype, and at each locus, when frequency of 1 genotype increases (e.g. paternal), the other genotype decreases proportionally (e.g. maternal). In the example loci described in Fig. 5b, we observed that 2 contigs increase in alternative allele frequencies, indicating an increase in maternal genotypes at that particular locus. However, for all heterozygous markers across the genome, the shifts in parental genotype frequencies are balanced, showing no overall preference for maternal or paternal alleles.

Previous studies have inferred that marine bivalves have a large genetic load (Launey and Hedgecock 2001; Plough 2016; Plough et al. 2016; Plough 2018; Österling et al. 2020; Durland et al. 2021). Larval development for most marine invertebrates is often a prolonged and complicated physiological process because larval survival is characteristically very low, with only a small proportion of individuals surviving from fertilization to the juvenile (spat) stage (type III survivorship) (Plough et al. 2016; Plough 2018). In our study, we described genetic changes taking place within a single generation of populations of mussels over 23 days of larval development. The effects of genetic load have been demonstrated to induce signatures of selection in highly fecund animals, such as plants and some marine bivalves (Turner and Williamson 1968; Launey and Hedgecock 2001), and high genetic load contributes to mass mortality events during early developmental stages for bivalves (Richard et al. 2021). One classic population genetics theory assumes that most genetic variation has a relatively small effect on fitness, and this variation could only be altered by selective pressures gradually over millions of years (Pespeni and Palumbi 2013). However, our study and others (Gosset et al. 2014; Bitter et al. 2019; Durland et al. 2021; Durland et al.2022) have challenged this and demonstrated the importance of standing genetic variation and how temporally variable selection forces could maintain significant levels of genetic variation. The majority of SNPs identified in our study did not show a tendency for selection toward either allele (i.e. directional or stabilizing selection), and thus it is difficult to assign qualitative traits (i.e. beneficial or deleterious). We hypothesize that the preservation of alternative alleles in a population through temporally variable selection improves chance of survival for *M. galloprovincialis* and that this may contribute to an above-average genetic load for this species and possibly for other marine bivalves. Furthermore, this phenomenon may be acting in other sessile, highly fecund marine broadcast spawners from other phyla which reproduce in hyper dynamic micro- or nano-environments.

As with survival, growth rate and terminal adult size are commercially important traits and perhaps selectable [bigger size is correlated with fast growth (Ernande et al. 2003; Rose et al. 2009)]. Revenue for most bivalve aquaculturists is dependent on farm yield, which can be considered a function of both growth rate and survival. It has been demonstrated in the Pacific oyster that yield-per-oyster can be improved by phenotypic selection [113], though some evidence also suggests that there is a trade-off between growth rate and survival in the Pacific oyster (Ernande et al. 2003). Our Fig. 3d shows a negative correlation between size and survival on day 23, which is consistent with the hypothesis that fast-growing larval cohorts may underperform in survival as compared to their slow-growing cohorts (Ernande et al. 2003), especially considering that between-tank environmental fluctuations (feed, temperature, pH, salinity, and dissolved oxygen) were minimal. We used significant changes in SNP frequencies between the 2 phenotypic groups, big and small, on day 23 to identify size-associated SNPs (Table 1, column 12, and Supplementary Table 9). Although there were some overlapping sites between size-associated SNPs and viability-associated SNPs, the majority of sites in this study were unique only to 1 trait (Table 2 and Supplementary Table 10). Our data show very few overlaps in viability-associated and size-associated SNPs. A recent paper has corroborated the idea that viability-associated SNPs are largely distinct from growth SNPs (Pan et al. 2023). The limited amount of overlap in size-associated loci and viability-associated loci in our data suggest possible genetic trade-offs between survival and growth, supporting results from previous research on the Pacific oyster which suggest that a selective breeding program to improve survival rates may not necessarily improve growth rates or that lines must be selected for each individual oceanic outplanting site (Ernande et al. 2003; Langdon et al. 2003). Similar correlations have been found in *Drosophila melanogaster* (Chippindale et al. 1997; Borash et al. 2000; Munch et al. 2003), but studies assessing the phenotypic and genotypic evidence for such relationships in bivalves remain scarce (Ernande et al. 2003; Langdon et al. 2003). In marine invertebrates, the early development period is extremely tenuous time, as it requires a high level of energy (Hrs-Brenko and Filic 1973; Ceccherellia and Rossi 1984; Rodrigez et al. 1990). Larger larvae may require more resources to maintain physiological homeostasis (Rodrigez et al. 1990) which could negatively impact their survival rates (Kingsolver and Huey 2008). It has been suggested that selective breeding programs should choose survival as a primary character to improve harvestable yield because the impact of large-scale, early mortality events are so detrimental to commercial endeavors (Dégremont et al. 2005). However, the growth and survival strategies in the early development of the Mediterranean mussel may change drastically when planktonic larvae become sessile juveniles/adults (Bassim et al. 2015). Because of their complex life cycle, bivalve growth rates and survival from metamorphosis and settlement to harvest demand more detailed assessments. Further comprehension of growth and survival at various developmentally important times is essential for improving yield and establishing broodstocks of Mediterranean mussels with desirable characteristics. As of the time of publication, the authors are attempting to develop ocean acidification-resistant lines of Mediterranean mussel which will undergo a similar genomic analysis to the study described here; it will be very interesting to investigate any SNP overlap between size, survival, and ocean acidification resistance.

## Supplementary Material

jkad103_Supplementary_Data

## Data Availability

The data sets generated during and/or analyzed during the current study are available in the rachelzh-hua/mussel_project repository, https://github.com/rachelzh-hua/mussel_project or available from the corresponding author on reasonable request. Supplemental material available at G3 online.
