# Supplementary material for: Balancing selection and candidate loci for survival and growth during larval development in the Mediterranean mussel, Mytilus galloprovincialis
Source: G3 (Bethesda). 2023 May 13;13(7):jkad103. doi: 10.1093/g3journal/jkad103 (PMC10320762; doi:10.1093/g3journal/jkad103)
Supplement: jkad103_Supplementary_Data [file jkad103_supplementary_data.zip › File_S1_G3-2022-403903.pdf]

Supplemental Documents

Bivalve DNA Extraction Protocol

Equipment Needed:

- Animals/Specimens
- Heat Station
- Liquid Nitrogen
- Zymo GDNA Extraction Kit
- Ice
- Qubit Machine & HS-DNA Reagents
- 4°C centrifuge (non 4C seems to work well also)
- Razor blades
- Pestle and Mortar
- Proteinase K
- Tissue and Cell Lysis Solution
- MPC Protein Precipitation Reagent
- Eppendorph pestle and pestle vibrating tool

Protocol:

Set Up:

1. **Create Lysis solution by adding 20 uL of Proteinase K into 300 uL of Tissue and Cell Lysis solution. If you anticipate more than one sample, multiply this equation**

accordingly. (i.e. two samples = 40 uL Proteinase K and 600 uL Tissue & Cell Lysis solution).

2. Turn Heat Station to 55 C
3. Get Ice
4. Get Liquid Nitrogen
5. Turn centrifuge on and make sure it is set to 4°C if applicable
6. Wipe down lab area with 70% EtOH

Grinding Animal Tissues (Skip this step if processing larval samples)

1. If frozen, get animal from -80C Freezer.
2. Cut an approximately 1 cubic cm (or less!) chunk from the animal. You want enough animal to fill an Eppendorph tube approx. ¼ way, too much will result in poor quality DNA. Place animal tissue not being used back into the freezer immediately.
3. Place animal tissue into mortar, fill mortar carefully with liquid Nitrogen, and grind. Place your hand around the pestle so that you don't send chunks of bivalve flying all over the place. Repeat this step as necessary adding liquid Nitrogen to keep tissues frozen. You must get the tissue ground to a FINE POWDER!

DNA Extraction

4. Place animal tissues into a 1.7 mL Maxximum Recovery tube, approximately ¼ to ½ way full. DO NOT OVERFILL!
5. Add 300 uL of Cell Lysis solution with Proteinase K to each tube.
6. Use Eppendorph pestle and pestle vibrating tool to homogenize tissue and solution as best you can. It may get very foamy and gooey, that's fine. Vortex. NOTE: if processing larval samples, use hand pestle to homogenize gently.

7. Incubate at 55 C for ~~at least 15 minutes,~~ 10 minutes, vortex every ~~5~~ 2 minutes.
8. Place the samples on ice for 3-5 minutes.
9. Add 150 uL of MPC protein Precipitation Reagent to each of your samples and vortex vigorously for at least 10 seconds. **Let stand for 1 minute.**
10. Pellet the debris by centrifuging at 4 C for 10 minutes at max speed.
    a. If the resultant pellet is clear (i.e. not there), small, loose, the liquid is extremely milky, etc., add an additional 25 uL of MPC Protein Precip Reagent, mix, and pellet again.
11. Transfer the supernatant only (i.e. the clear liquid) to clean 1.7 mL Maxx Recovery tubes in 200 uL aliquots (discard the protein pellet).
12. Start with step 1 of the Zymo G-DNA extraction kit on the "Cell Suspensions and Proteinase K Digested Samples" protocol. For the first step, add 800 uL of Genomic Lysis Buffer to each tube.
    a. The protocol says that each filter only holds approx. 800 uL, but really they can hold approx. 1 mL
13. After completing the DNA Extraction kit, quantify DNA concentration with Qubit.
14. Record concentrations, label tubes, and place DNA in -20 C freezer (or on ice if you are planning to use immediately).

Library Generation Protocol: Bivalve Specific ddRAD

PREPARING ddRAD LIBRARIES

Original Written Protocol: Wendy Vu

Bivalve Specific Protocol: Nathan Churches

NOTES: This protocol is for generating a small number of libraries at a time, and has been optimized for mussel ddRAD seq using SphI-HF and Mlu-CI Restriction Enzymes. This process can be streamlined for 96 well plates by referencing Wendy's original protocol. It is assumed that you know how to quantify DNA using Qubit and clean using MagBio beads for this protocol.

1) Annealing Protocol for Adapters

Description: In this step, you are taking the single stranded adapters that you purchased (both the barcoded and common adapter) and annealing them so that they are double stranded. These will later go through the restriction digestion process, and be bound with digested DNA. Before starting, you need to adjust the molarity of the single stranded adapters to 200 uM. This is typically done by adding the appropriate amount of water at the onset into the dry tube that the single stranded DNA comes in.

Protocol:

In a PCR Tube bring together:

- Top strand DNA oligo (200uM starting concentration) 5ul
- Bottom strand DNA oligo (200uM starting concentration) 5ul
- TE 10ul (Tris-EDTA, pH 8.0, can be purchased via Fisher)

Total volume 20ul

Primers are 50uM after dilution.

In Thermocycler:

- 95 degrees for 2min
- Ramp to 25 degrees by 0.1 degree per second
- Hold at 25 degrees for 30 min
- Hold at 4 degrees forever

Plus/Minus Check for Annealing

To check the performance of the annealing, run 1ul of annealed adapters side by side with 1ul of un-annealed adapter in a 4% agarose gel. Sybrsafe does not bind to single stranded DNA, so the un-annealed adapter lane will look blank (However, when using Safeview, we observed that the intensity of the un-annealed adapter band was lower than the annealed adapters and also the size was smaller). The annealed adapters should show up at ~45bp.

Making 4% Agarose gel with Safeview:

- Weigh the agarose and put it in a bottle with 1X TBE buffer overnight to hydrate the agarose
- Next morning: heat the agarose in the microwave for 2 min (check during heating to avoid spilling out). Check while heating to observe any floating debris, which is undissolved agarose.
- Add 5ul of Safeview to 100 ml agarose gel, stir, and pour immediately very carefully.

2) Adapter Working Solutions

Description: In the following steps, you will take the adapters, which are now double stranded and stable at a concentration of 50 uM, and create dilutions that you can work with in the lab. *Water can work in place of TE.

First Dilution (5uM)

To make working solutions, bring together:

- 5ul of 50uM stock solution of annealed adapters
- 45ul of 1X TE*
- Vortex and spin down

Total Volume: 50ul

Second Dilution, Working Adapter stock (0.5uM)

Dilute the above stock mix 1:10 stock:water

Use Qubit to quantify your working adapter stock. Concentrations should be approximately 60 ng/uL.

NOTE!: The ending concentration is the most important, not the dilution. Ensure you get to ~60ng/uL.

3) First restriction digestion and ligation of sample DNA and Barcoded Adapter

Description: In this step, you will digest your DNA using the first RE: SphI-HF. The restriction enzyme digestion is immediately followed by ligation, and so the barcoded adaptors (as opposed to the common adapter) are included into the restriction digestion but won't be affected since they do not have the restriction enzyme recognition sites. If you change the REs used in this protocol, you must go back in and ensure your adapters do not have any sites that match the RE cut site. You should have high molecular weight DNA before this process. This protocol may not be as effective if you are using degraded DNA.

Protocol:

- In a PCR tube, add enough template DNA for a concentration of 100 ng of DNA in 10 uL of water.
- Add 2 uL of the barcoded adapter you intend to use for each sample, which should total approximately 120 ng of adapter.**

  **This ratio of [120ng:100ng adapter:DNA] can be optimized for different genomes and adapter sets, but works well for Mytilus galloprovincialis.

Digestion 1 with SphI-HF

Mix for First Digestion	uL
DNA:Adapter Mix	12
H20	5
CutSmart Buffer	2
SphI-HF (20U/uL)*	1
Vol final	20

Incubate 37C for 1 hour

Heat at 65C for 20min (inactivation)

*Consult the card that comes with the RE and adjust this table accordingly. This worked as of 2018 for SphI-HF.

Ligation 1

Mix for Ligation 1	uL
Digested DNA&Adapter, prev step	20
H20	23.4
T4 Buffer	5
T4 ligase (400U/uL)	1.6
Vol final	50

Incubate 22C for 1 hour

Heat at 65C for 10 min (inactivation)

Clean using MagBio beads at 1.6X, to rid mixture of small leftover adapter molecules (1.6X * 50 = 80 uL MagBio). Elute in 40 uL of water, and collect 37 uL for the next step. (Leaving 3 uL ensures no bead contamination for the next steps).

4) Second restriction digestion and ligation reaction using Common Adapter

Description: In this step, you will repeat the process as in step 3, but with MLU-CI restriction enzyme and the common adapter instead. After cleaning (step 5), you will have a 'pre-library' that is ready for PCR enrichment.

Protocol:

Digestion 2 with Mlu-CI

Mix for First Digestion	uL
DNA From Step 3	37
Common Adapter	4
CutSmart Buffer	4
Mlu-CI (20U/uL)*	1
Vol final	46

Incubate 37C for 1 hour

Heat at 65C for 20min (inactivation)

*Consult the card that comes with the RE and adjust this table accordingly. This worked as of 2018 for Mlu-CI.

Ligation 2

Mix for Ligation 2	uL
Digested DNA&Adapter, prev step	46
T4 Buffer	5
T4 ligase (400U/uL)	1
Vol final	52

Incubate 22C for 1 hour

Heat at 65C for 10 min (inactivation)

5) Library Size Selection

Description: In this step, you will take your libraries and size select for smaller fragments, which are required for effective PCR. This is done using the properties of MagBio beads.

Protocol:

- Clean using MagBio beads at 1.6X, to rid mixture of small leftover adapter molecules (1.6X * 52 = 83.2 uL MagBio). Elute in 40 uL of water, and collect 37 uL for the next step. (Leaving 3 uL ensures no bead contamination for the next steps).

- Add .5X MagBio beads to cleaned DNA (37 * .5 = 18.5 uL), and recover supernatant on step 1 of MagBio Protocol. This should yield ~ 55 uL of small fragments only (37 + 18.5 = ~55). You may continue with the MagBio Protocol with the larger fragments that are stuck on the beads, if you want to test for efficacy of size selection at later steps. The large fragments may be tossed out if unneeded.
- Using the 55 uL, clean again at the full 1.8X MagBio Beads (55* 1.8X = 99 uL MagBio). Run through entire MagBio Protocol, eluting in 40 uL water and recovering 37 uL.
- This is now your size selected 'pre-library', ready for PCR.
- Quantify using Qubit.

6) PCR enrichment

Description: You should have only small fragments in your 'pre-library', which are now at fairly low concentrations. PCR will help enrich the concentration, and this step also adds the illumina platform sequences via the primers. After PCR, you'll have fragments of DNA in the size range of approximately 100-1000 bp with a [barcode+illumina_index] on one end, and a [common_adapter+illumina_index] on the other. This is your library, ready for the BioA! You can perform several PCR reactions per library in order to increase the concentration of library. For example, Wendy had 12 PCR reactions per library, resulting in 600 uL of total amplified library. This can then be cleaned and concentrated for higher concentration libraries.

Mix for PCR	uL

DNA Pre-Library	See Note
Water	Fill to 50
5x HF buffer	10
2.5uM dNTPs	5
P1 primer (5uM starting)	4
P2 primer (5uM starting)	4
MgCl2	2
Phusion HF Taq	0.5
Vol final	50

NOTE: ~~It is CRITICAL that you have a ratio of [25ngDNA:2uMPrimer]. 2uM represents the final concentration of the primers, which are starting at 5uM. Use the equation C1V1=C2V2 to determine appropriate amount of DNA Pre-Library to add. For example, if you had a pre-library at a concentration of 20ng/uL, I'd do the following:~~

~~C1*V1=C2*V2~~

~~(20ng/uL)*V1=(2uM)*(50 uL)~~

~~V1 = [(2uM)*(50 uL)]/(20ng/uL)~~

~~V1 = 5 uL of pre-library to add~~

**NOTE: The above strike-through was wrong somewhere…On 1/28/19 through 2/2/2019 I underwent a BioA optimization of the appropriate ratio and cycling of primers for PCR. The result shows that for every 1 uL of primer at 5 uM, you need approx. 2ng of DNA. SO, add up to 8ng of your library to achieve the appropriate balance for 4 uL of 5uM primer. EX:

DNA total ng	Primer volume to add (at 5uM start conc.)
2	1
3	1.5
4	2
5	2.5
6	3
7	3.5
8	4

Amplify using the following PCR protocol:

98C 30sec

10 cycles of:

98C 10sec

65C 45sec

72C 15sec

72C 2min

Hold at 4C

NOTE: Reducing the number of cycles may reduce amplification of bigger fragments >600bp, which will not be sequenced efficiently by Illumina sequencing. 10 cycles was found best given the 1/28/19 optimization trial.

Optional: Pool PCR reactions if you have performed multiple reactions.

-Clean PCR reactions using MagBio Beads at 1.6X, then quantify PCR reactions using Qubit.

-Test Libraries using BioAnalyzer and qPCR.

-Sequence!
