# Supplementary figures and images for: Balancing selection and candidate loci for survival and growth during larval development in the Mediterranean mussel, Mytilus galloprovincialis
Source: G3 (Bethesda). 2023 May 13;13(7):jkad103. doi: 10.1093/g3journal/jkad103 (PMC10320762; doi:10.1093/g3journal/jkad103)

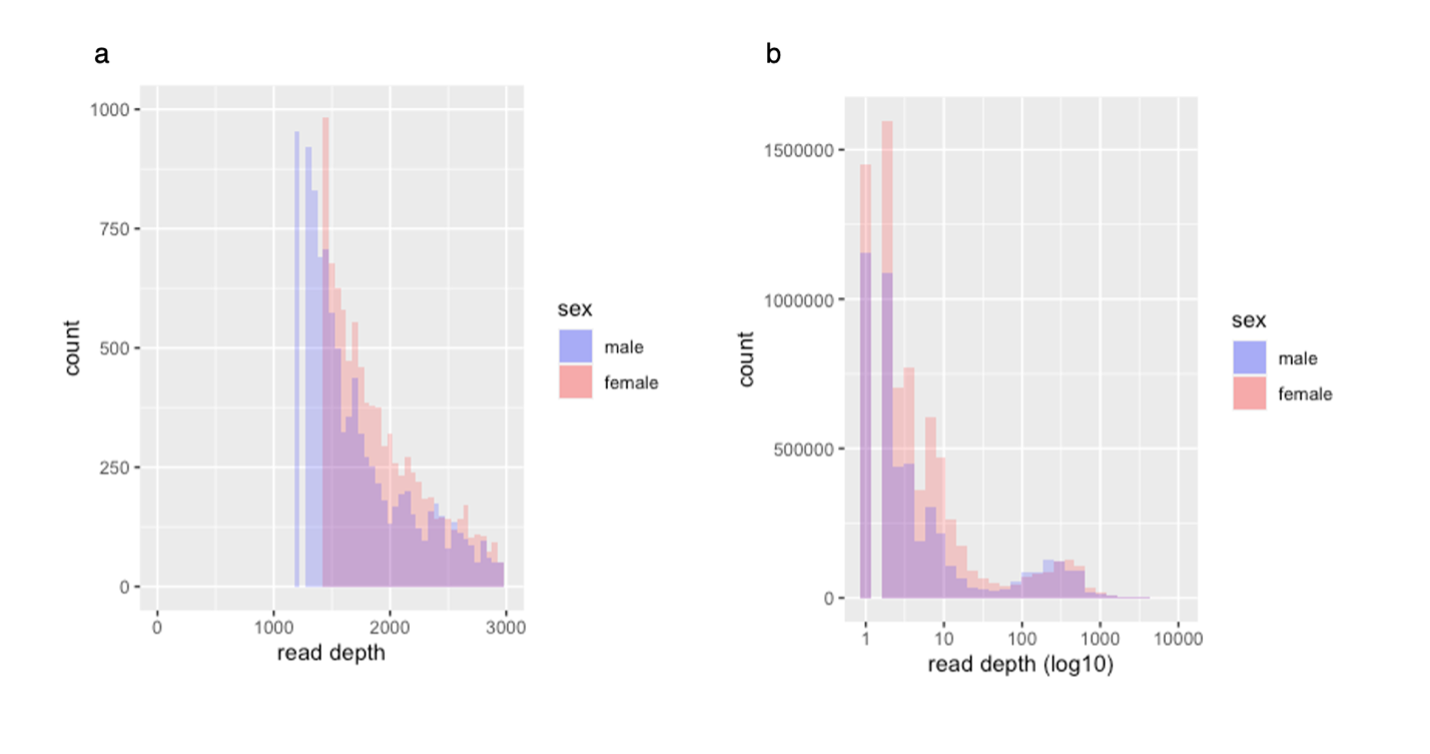

Supplement: jkad103_Supplementary_Data [file jkad103_supplementary_data.zip › Figure_S1_G3-2022-403903.png]

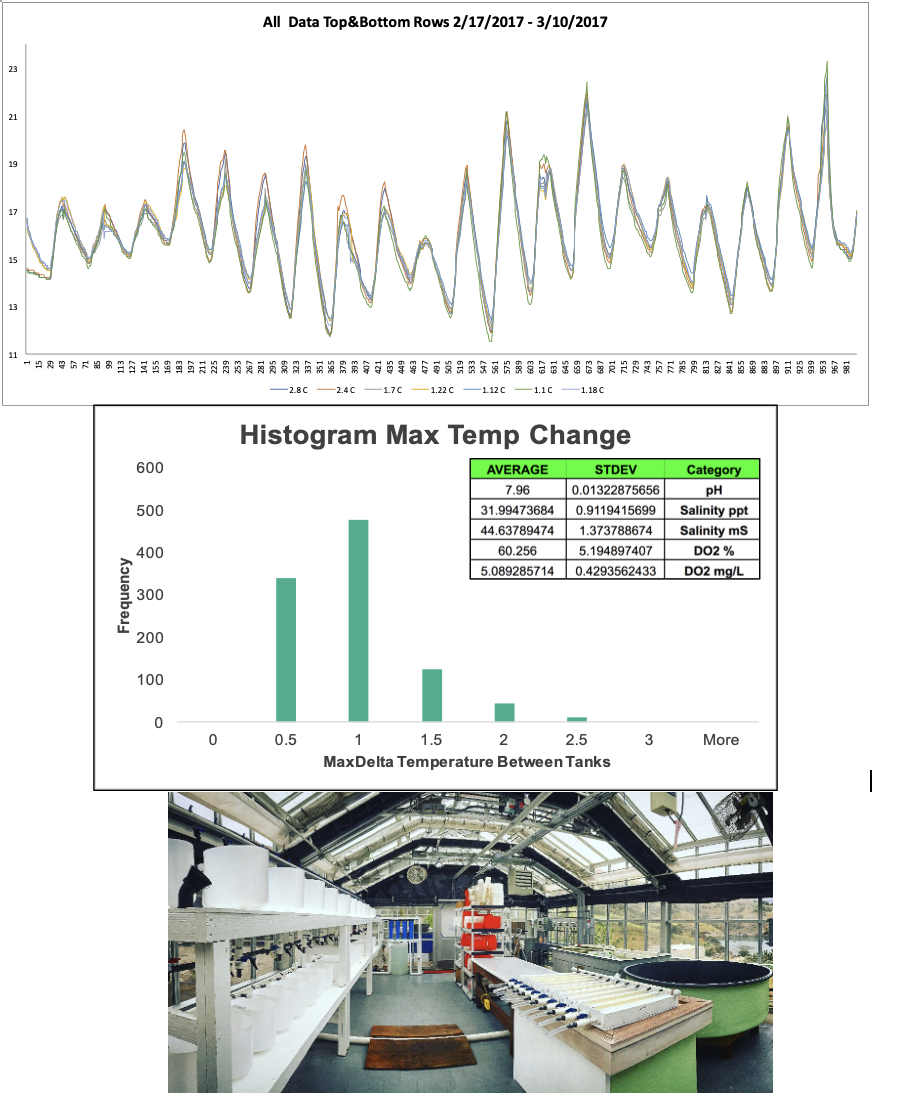

Supplement: jkad103_Supplementary_Data [file jkad103_supplementary_data.zip › Figure_S2_G3-2022-403903.png]

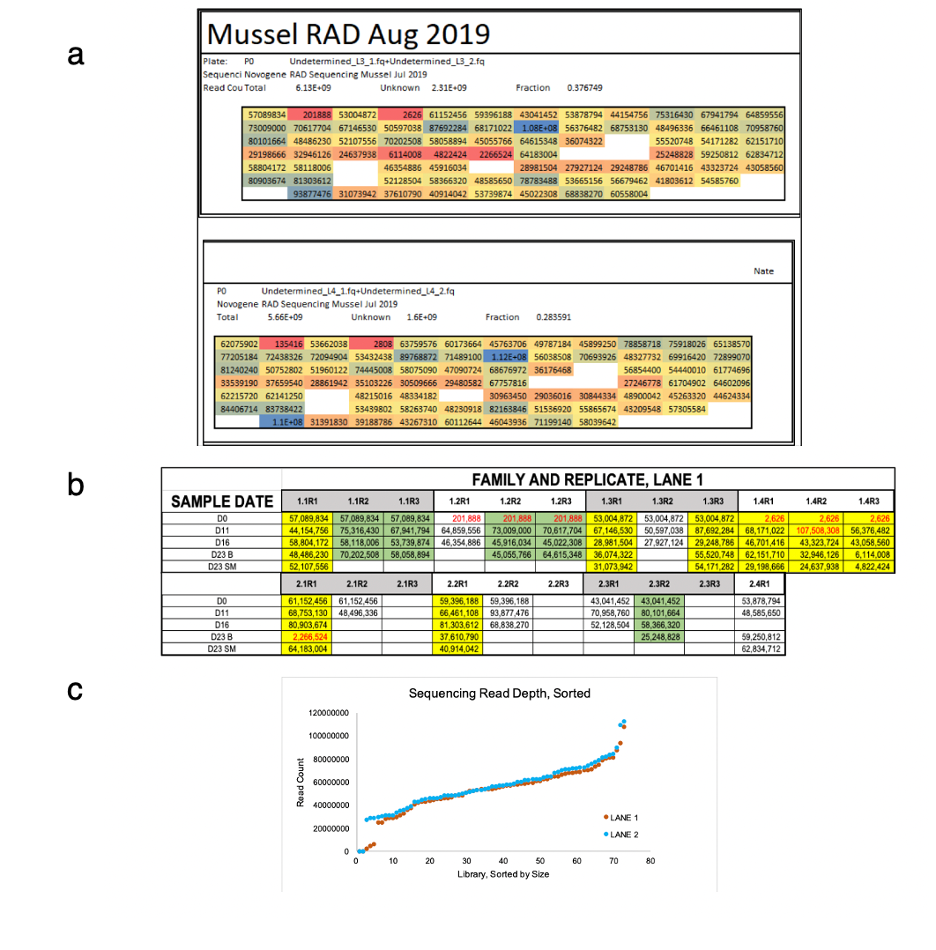

Supplement: jkad103_Supplementary_Data [file jkad103_supplementary_data.zip › Figure_S3_G3-2022-403903.png]

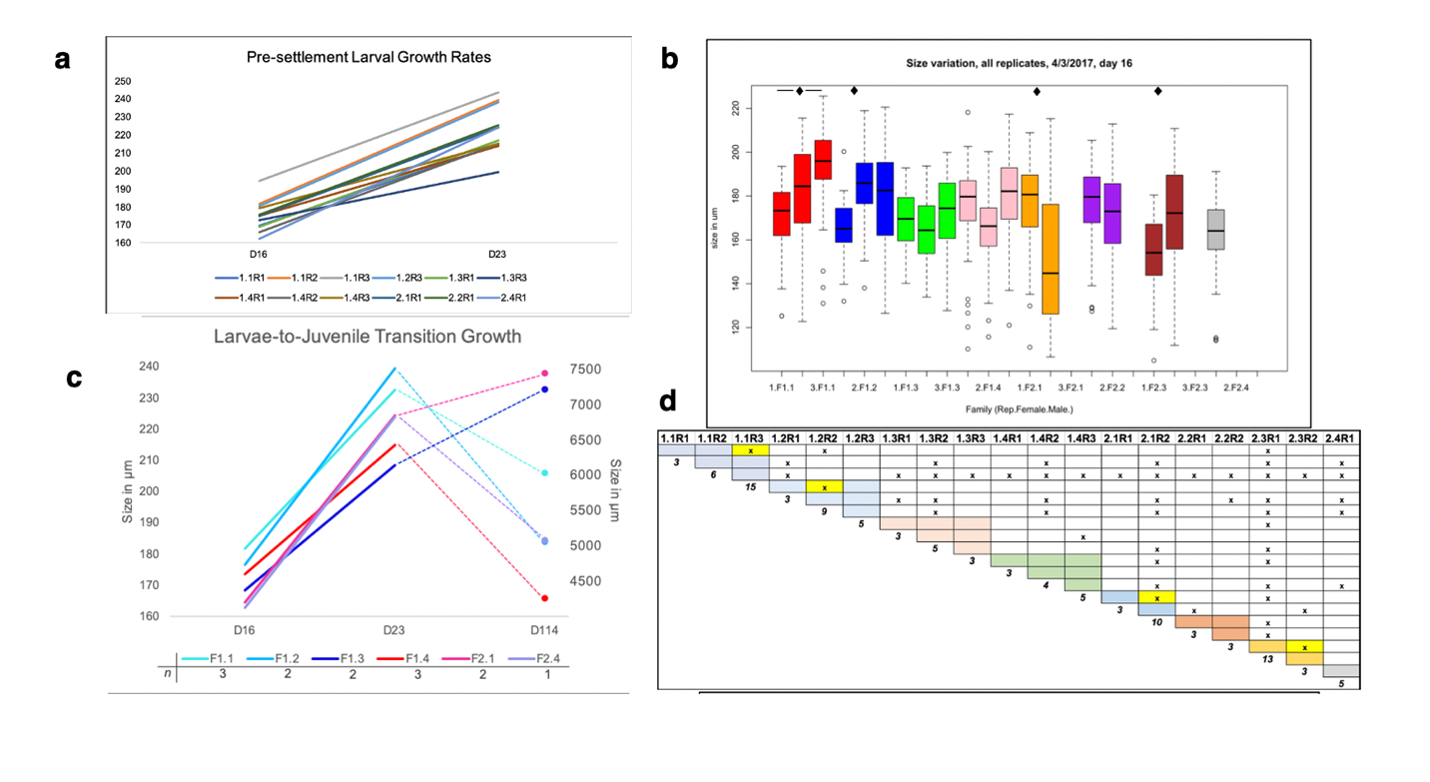

Supplement: jkad103_Supplementary_Data [file jkad103_supplementary_data.zip › Figure_S4_G3-2022-403903.png]

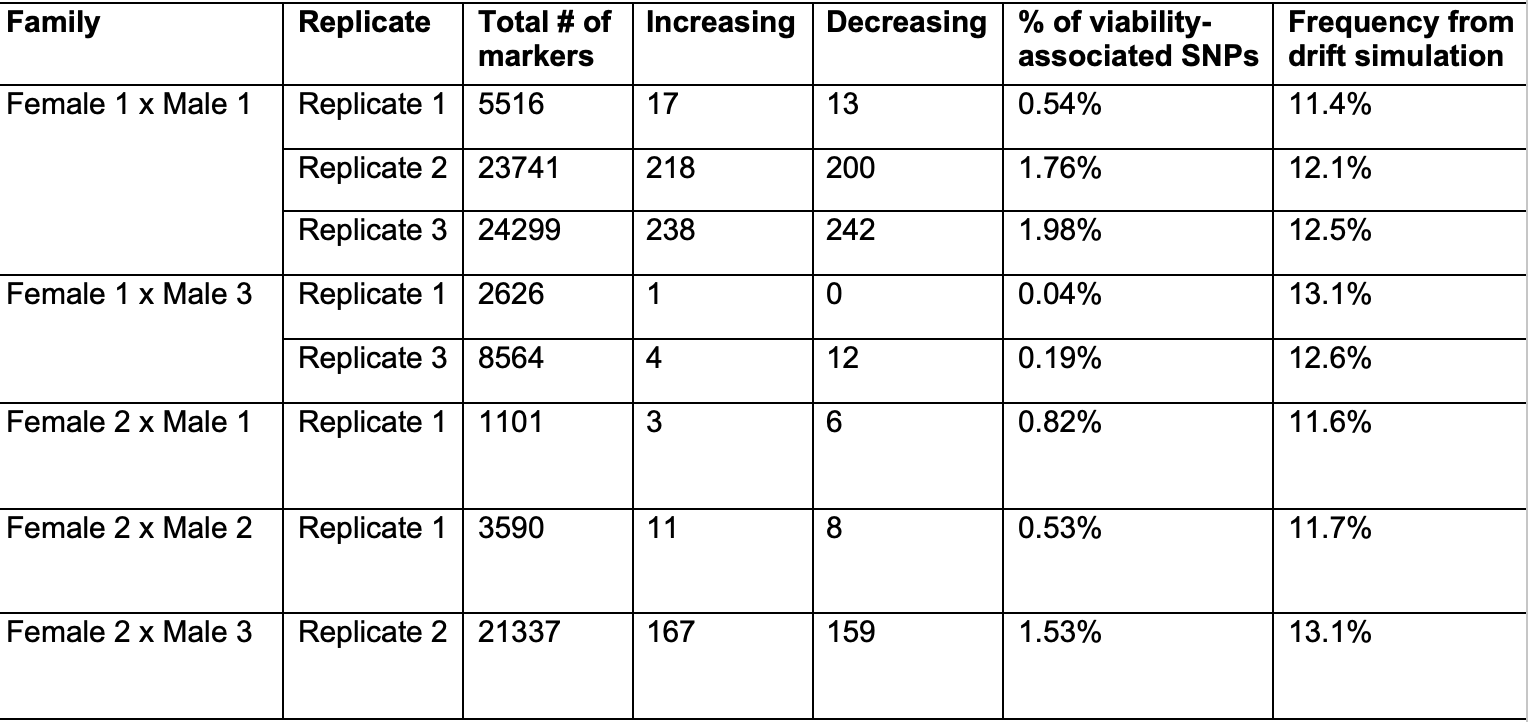

Supplement: jkad103_Supplementary_Data [file jkad103_supplementary_data.zip › Table_S11_G3-2022-403903.png]
